# Ancestry-Specific DNA Damage Repair Gene Mutations and Prostate Cancer

**DOI:** 10.3390/cancers17040682

**Published:** 2025-02-18

**Authors:** Talaibek Borbiev, Kevin Babcock, Kayleigh Sinopole, Gregory T. Chesnut, Gyorgy Petrovics

**Affiliations:** 1 Center for Prostate Disease Research, Murtha Cancer Center Research Program, Department of Surgery, Uniformed Services University of the Health Sciences, Bethesda, MD 20814, USA; tborbiev@cpdr.org (T.B.); gregory.t.chesnut.mil@health.mil (G.T.C.); 2 Henry M. Jackson Foundation for the Advancement of Military Medicine, Inc., Bethesda, MD 20817, USA; 3 Internal Medicine, Alexander T. Augusta Military Medicine Center, Fort Belvoir, VA 22060, USA; kevin.r.babcock.mil@health.mil; 4 School of Medicine, Uniformed Services University of the Health Sciences, Bethesda, MD 20814, USA; kayleigh.sinopole@usuhs.edu

**Keywords:** prostate cancer, DNA damage repair genes, germline mutations, somatic mutations

## Abstract

This review is intended to reflect the currently available literature on both clinically significant germline mutations in DNA damage repair (DDR) genes as well as the importance of ancestral diversity in the pathogenesis of prostate cancer (PCa). The second most prevalent cancer worldwide in men is PCa, causing significant morbidity and mortality in its advanced stage. Emerging data highlight the substantial role of germline mutations of DDR genes in PCa pathogenesis, especially in progression to aggressive forms of the disease. Germline genetic testing is recognized as a necessary tool for efficient, individualized patient care. NCCR guidelines recommend inquiring about the family history of PCa and known germline variants and, if indicated, proceeding with germline multigene testing followed by post-test genetic counseling. Depending on the germline mutations in HR repair genes or in MMR genes, specific treatment options may provide clinical benefit. We will discuss specific germline mutations that are involved in PCa progression and prognosis in racially diverse populations.

## Simple Summary

Prostate cancer (PCa) is a heterogenous malignancy in men, representing the second most diagnosed cancer and the fifth leading cause of cancer-related death globally in 2022. PCa incidence and progression is dependent on several predisposing factors, most significantly genetics and germline/somatic mutations. With the development of whole-genome sequencing (WGS) technology, it is now possible to determine the mutational statuses of PCa-related genes for improved clinical decision making and individualized patient care. The National Comprehensive Cancer Network (NCCN) Guidelines now recommend germline multigene testing followed by post-test genetic counseling. Depending on the germline mutations in homologous recombination (HR) repair genes or in mismatch repair (MMR) genes, specific treatment options, including poly-ADP-ribose polymerase (PARP) inhibition or PD-1 blockade, may be of more clinical benefit. This review highlights specific PCa-related germline mutations in racially diverse populations and therapeutic approaches to treat PCa patients with DNA damage repair gene alterations in advanced disease.

## 1. Introduction

PCa is the second most diagnosed cancer in men globally, with nearly 1.5 million new cases and 397,000 deaths, and was the fifth leading cause of cancer death among men in 2022 worldwide [1]. In the United States, PCa is the leading cancer diagnosis and the second deadliest cancer among men, with incidence estimated at 299,010 new cases and 35,250 expected deaths in 2024, with the survival rates dropping substantially for distant PCa when compared to localized or regional disease [2]. The risk for PCa occurrence and progression depends on several predispositions including an older age, with a median age at diagnosis of 67 years in the United States; family history; and ancestry, with non-Hispanic black men leading both new cases as well as mortality in the United States and globally [3]. Among 36 nations investigated, countries in Africa reported the highest mortality rates associated with PCa [4]. The risk factors of family history and race imply the substantial role of genetics and germline mutations in the pathogenesis of PCa.

The introduction of next-generation sequencing (NGS) has revolutionized genomic research, allowing WGS in a much shorter time, with multiple platforms and applications available [5,6]. The comprehensive knowledge of germline and somatic mutations in PCa promotes precision medicine opportunities. The mutations and genetic variants detected by NGS are useful for disease diagnosis, prognosis, and therapeutic interventions, heralding the era of precision medicine and individualized patient care. NGS makes possible germline and somatic genetic testing in clinical practice. However, existing technical, economic, and other limitations need to be addressed before it becomes the standard of care [7].

It is recognized that the incidence of PCa is markedly higher in patients of African ancestry (AA), often manifesting in a more aggressive and lethal form. One of the explanations for these disparities is the presence of genomic variations. Germline susceptibilities specific to various ancestral groups have been shown through genome-wide association studies, suggesting differential genomic architecture [8]. For instance, AA men display a genetic PCa risk score approximately 2.18 times higher than men of European ancestry (EA) whereas men of East Asian ancestry exhibit a risk 0.73 times lower than those of EA [9].

The need for a systematic understanding of germline and somatic mutations in cancers has started to propel opportunities in precision medicine. Genetic testing for inherited mutations, including DDR genes, has been rapidly increasing. The implementation of germline testing in the selection of PCa treatment, management, and hereditary disease assessment was a focus at the Philadelphia PCa Consensus Conference in 2019: large germline panels and somatic testing were recommended for metastatic PCa, with priority given to *BRCA1*/*BRCA2*, *ATM*, and mismatch DNA repair genes [10]. According to the latest NCCN Guidelines for PCa, germline multigene testing that includes at least *BRCA1*, *BRCA2*, *ATM*, *PALB2*, *CHEK2*, *HOXB13*, *MLH1*, *MSH2*, *MSH6*, and *PMS2* is recommended [11]. *BRCA1*-, *BRCA2*-, and *PALB2*-encoded proteins are tumor suppressors involved in the HR repair pathway of double-stranded DNA damage. ATM and CHEK2 are cell cycle checkpoint kinases while HOXB13 is a transcription factor highly expressed in the prostate. Encoded proteins of *MLH1*, *MSH2*, *MSH6*, and *PMS2* play an important role in DNA mismatch repair.

DDR and MMR genes are essential in repairing the continuously occurring spontaneous mutations, thereby preventing mutation-based cancer development. Emerging data highlight the role of DDR genes in localized and aggressive forms of PCa. An inactivating mutation in DDR genes may lead to mutation accumulation and potentially tumorigenesis and progression. DDR genes repair DNA double-stranded breaks through HR using sister chromatids as a template, restoring the original DNA sequence. Crucial proteins involved in mediating HR repair include those encoded by *BRCA1*, *BRCA2*, *ATM*, and *PALB2* [12,13]. The inability to efficiently repair DNA damage due to mutations in DDR genes is known as homologous recombination deficiency (HRD). The HRD tumor phenotype is sensitive to PARP inhibitors, such as the currently recommended olaparib, rucaparib, niraparib, and talazoparib, allowing the selective targeting of tumor cells harboring DDR-deficient genes in clinical settings of progressed PCa [11,14,15,16,17].

DNA mismatch repair is a conserved cellular process essential to maintaining genomic stability by repairing mismatched base pairs generated during DNA replication and recombination. Germline mutations in the DNA MMR genes *MLH1*, *MSH2*, *MSH6*, and *PMS2* are associated with Lynch syndrome. Defects in this cellular mechanism will result in genomic instability via a microsatellite hypermutator phenotype and a predisposition to the development of multiple cancers including PCa [18,19,20,21,22,23].

Several types of cancer immunotherapy have become valuable tools in the management of specific tumors. Among them, the inhibition of immune checkpoint blockade through programmed cell death 1 (PD-1) and its ligand (PD-L1) is considered to be a common approach for cancer therapy [24]. The binding of the inhibitory PD-1 receptor on T-cells to its ligand, PD-L1, on the surfaces of cancer cells leads to T-cell inactivity and immunosuppression. Multiple cancers can upregulate PD-L1 expression, thus escaping host immune defense mechanisms. PD-L1 expression is positively correlated with a deficiency in DNA MMR genes and microsatellite instability in cancer cells and is implicated in metastatic CRPC [25,26], making it possible to use PD-1/PD-L1 inhibitors in order to expose tumor cells to host immune attack.

In this review article, the most common germline mutations in DDR and DNA MMR genes implicated in the pathogenesis of PCa within genetically diverse populations will be discussed, as will existing therapies for patients with these mutations.

## 2. Germline Mutational Spectrum and Ancestral Disparity in PCa

PCa occurrence and mortality rates are notably affected by ancestral disparities. Regardless of current progress in PCa diagnosis and therapeutic approaches, AA men are known to carry a higher risk of advanced and metastatic disease. Out of about 300,000 new PCa cases recorded annually in the USA, an estimated 42,000 new cases, or 37%, occur in AA men, bringing the average annual PCa incidence rate to 172.6 cases per 100,000 in AA men, 73% higher than the rate in EA men [27]. Also, AA men have the highest death rate from PCa of any ethnic group in the USA, two times higher than EA men, attributed to the more aggressive pathogenesis of PCa in AA men [28]. Although the disparity in mortality may be reflected by disparities in access to care, genetics and the differential mutational burden of PCa-related genes may be significant contributing factors [29]. Most of the research to identify pathogenic/likely pathogenic (P/LP) variants was conducted in EA patient cohorts even though there are indications of significant ancestral differences in the mutation rates, stressing the need to expand studies in diverse populations.

In a multi-ancestry genome-wide association study of 156,319 PCa cases, with contribution from our Center for Prostate Disease Research, the genetic risk score for aggressive PCa versus nonaggressive PCa was higher in AA men [30], reflecting ancestral differences in diverse populations. Microarray-based methods measuring expression levels of 517 PCa-associated genes in 639 tumor specimens from AA men (270) and EA men (369) demonstrated distinct groups of 95 genes overexpressed in AA men and 132 genes overexpressed in EA men [31], supporting the role of existing genetic differences between diverse populations in the pathogenesis of PCa.

The importance of mutations in DNA repair genes was shown in a study of 200 largely EA men (66.5%), where 63.6% of mutations in 28 cancer-related genes were in DDR and DNA MMR genes [32]. Utilizing a cancer-related panel of 30 genes associated with hereditary PCa risk in 1351 patients (78% EA men), it was shown that most prevalent P/LP variants were in the *BRCA2*, *CHEK2*, *MUTYH*, and *ATM* DDR genes [33]. In a study of 1878 PCa patients (70% EA men) undergoing PCa multigene panel testing, the rate of *BRCA1* and *BRCA2* mutations was 4.6%, and the rate of mutations of the DNA MMR genes was 2.8% [34], supporting the genetic testing approach for hereditary PCa. In a systematic search of scientific literature for germline mutations, familial predispositions, and clinical implications in PCa, from 2000 to 2022, germline mutations in HR genes (*BRCA1/2*, *ATM*, and *CHECK2*), in MMR genes (*MLH1*, *MLH2*, and *MSH6*), and other additional genes were found to be involved in the pathogenesis of PCa and progression to metastasis [35].

While some variants of germline mutations in PCa of AA men are known, elevated levels of variants of unknown significance (VUS) in comparison with EA men also occur [36], underscoring the need for further genomic research in diverse populations. To determine the germline mutational landscape in AA and EA men, a 14-gene panel (*ATM*, *BRCA1*, *BRCA2*, *CHEK2*, *EPCAM*, *HOXB13*, *MLH1*, *MSH2*, *MSH6*, *NBN*, *PALB2*, *PMS2*, *RAD51D*, and *TP53*) was used in 427 patients: 237 AA (56%) and 190 EA (44%) men. Overall, AA men showed lower rates of pathogenic or likely pathogenic (P/LP) variants than EA men (5.91% vs. 11.05%, *p* = 0.05). The P/LP spectrum was narrower in AA men for the *BRCA2*, *PALB2*, *ATM*, and *BRCA1* genes. In addition, a significant difference was noted in the rates of VUS between AA and EA men, 25.32% vs. 16.32% (*p* = 0.02) [37]. In a study of AA (214 patients) and EA (2488 patients) men with PCa, patients were evaluated for P/LP variants in the DNA repair genes *BRCA2*, *BRCA1*, *PALB2*, *ATM*, *RAD51C*, *CHEK2*, *PMS2*, *BARD1*, *BRIP1*, *MLH1*, *MSH2*, *MSH6*, *NBN*, and *RAD51D*. Similarly, P/LP variants were less likely to be present in AA men and such variants were not detected within the *BARD1*, *BRIP1*, *MLH1*, *MSH2*, *MSH6*, *NBN*, and *RAD51D* genes in this group [38]. In a study investigating the scope of DDR gene germline mutations in 3607 PCa patients from diverse populations, it was found that 17.2% had a positive P/LP variant as follows: *BRCA2*, 4.74%; *CHEK2*, 2.88%; *ATM*, *2.03%*; *MUTYH*, *2.37%*; *APC*, 1.28%; *BRCA1*, 1.25%; and *PALB*2, 0.56%. The incidence of P/LP variants in the DNA MMR genes *PMS2*, *MLH1*, *MSH2*, and *MSH6* was lower, occurring in 1.74% of the study population. AA men in comparison with EA men had lower rates of positive variants [39]. Overall, AA men with PCa show lower incidence in P/LP variants in HR and DNA MMR genes while expressing more VUS than EA men. Elevated rates of VUS in AA patients warrants extended genomic research to ensure individualized patient care across diverse populations.

In metastatic PCa and castration-resistant prostate cancer (CRPC), evidence shows greater incidence in germline and somatic mutations of HR repair and DNA MMR genes. To explore the incidence and outcomes of somatic and germline mutations in HR repair genes, 729 patients with metastatic PCa, mainly of EA, were divided into a *BRCA1/2* mutation group, a group with HR mutations except *BRCA1/2*, and a non-HR group. Patients were tested for mutations by NGS for the following genes: *ATM*, *BRCA1*, *BRCA2*, *BRIP1*, *CDK12*, *CHEK2*, *FANCA*, *HDAC2*, *PALB2*, *RAD51B*, and *RAD54L*. Of 729 patients, 13.2%, 17.4%, and 69.4% were in the *BRCA1/2*, non-*BRCA*, and non-HR groups, respectively. Patients with *BRCA1/2* mutations had notably worse outcomes than non-HR or non-*BRCA* patients (*p* < 0.05), and progression-free survival and overall survival were remarkably shorter for BRCA than HR or non-BRCA patients (*p* < 0.05), suggesting a role for appropriate genetic screening to improve PCa prognosis [40].

Pathogenic/likely pathogenic/deleterious (P/LP/D) germline variants in DDR genes and their correlations with aggressive PCa or indolent disease were tested in a patient cohort of 5545 EA men (2775 indolent and 2770 aggressive, including 467 metastatic PCa cases). *BRCA2* and *PALB2* had the most statistically significant associations, with 2.5% of aggressive and 0.8% of nonaggressive cases carrying P/LP/D *BRCA2* alleles (odds ratio [OR] = 3.19) and 0.65% of aggressive and 0.11% of nonaggressive cases carrying P/LP/D *PALB2* alleles (OR = 6.31). *ATM* had a nominal association, with 1.6% of aggressive and 0.8% of nonaggressive cases carrying P/LP/D *ATM* alleles (OR = 1.88). DDR gene variants were more common in patients with advanced PCa than in patients with nonaggressive cases (14.2% vs. 10.6%; *p* = 5.56 × 10^−5^). Within DNA repair genes, most P/LP/D alleles were found in *BRCA2*, *PALB2*, and *ATM* [41]. In a study of 692 patients, mainly EA (83% EA men, 6% AA men, and 11% others)**,** metastatic PCa tumors were positive for P/LP in 11.8% vs. 4.6% in localized PCa (*p* < 0.001). Mutations were found in 16 genes including *BRCA2* (5.3%), *ATM* (1.6%), *CHEK2* (1.9%), *BRCA1* (0.9%), *RAD51D* (0.4%), and *PALB2* (0.4%) [42]. In a meta-analysis of largely EA patients, who were diagnosed with advanced PCa progressing to metastatic or PCa-related death, mutation rates in the *NBN*, *BRCA2*, *ATM*, *CHEK2*, and *PALB2* (*p* < 0.05) genes, with corresponding ORs of 6.38, 3.41, 1.93, 1.53, and 2.63, were notably higher than in nonaggressive PCa patients [43]. In a metastatic patient cohort (CA men, 89%), P/LP variants were identified in *CHEK2* (3.1%), *BRCA2* (2.7%), *ATM* (1.1%), *NBN1* (0.5%), *BRCA1* (0.4%), *PALB2* (0.4%), *PMS2* (0.4%), and *MSH6* (0.2%), with a total mutation rate of 8.7% [44]. Overall, germline and somatic mutation rates in HR repair and MMR genes were higher in progressed PCa in men with European ancestry.

While the majority of PCa research studies detecting P/LP variants of germline or somatic mutations have been performed in EA men, the frequencies and distribution of germline mutations vary in divergent populations. Therefore, it is important to extend related studies to PCa patients from diverse ancestries. The mutation rates of DNA repair genes in advanced and metastatic PCa patients of African ancestry were studied in a patient cohort of 188 AA men and 669 EA men. Using clinical panels, which included up to 86 cancer-related genes, no significant difference in P/LP variants was shown between AA and EA patients, except for *BRCA1* P/LP variants, which were higher in the AA group (OR of 4.86, *p* = 0.04). However, higher levels of VUS were demonstrated in AA patients compared with EA patients: 55.3% vs. 36.6%, respectively [45]. The WGS of advanced PCa cases (45 progressed tumors vs. 11 unmatched non-tumors) in patients of African ancestry showed that in comparison with TCGA-based data for EA men, AA men had higher levels of mutations in the BRCA2 (27%), APC (20%), ATM (20%), BRCA1 (13%), DNAJC6 (13%), EGFR (13%), MAD1L1 (13%), MLH1 (11%), and *PMS2* (11%) genes [46].

In addition, prostate tumors within patient populations of African ancestry seem to have an elevated tumor mutational burden. In a study, the WGS of high-risk prostate tumors in patients of African ancestry showed a 1.8-fold increase in somatic variants in comparison with PCa patients of European ancestry. Copy numbers of *CCND1* and *MYC* were commonly gained at later stages of PCa progression. PCa patients of African ancestry had lower levels of *PTEN* mutations and no *ERG* fusions or *PIK3CA* mutations when compared to patients of EA [47]. Furthermore, in the WGS analysis of 180 (115 of AA and 61 of EA) mainly advanced prostate tumors, with Gleason scores of 8 or higher, the spectrum of structural variations (deletions, duplications, inversions, translocations, and others) was two times higher in those of AA. Somatic hotspots affecting 18 new possible PCa driver genes, including *CADM2*, *LSAMP*, *PTPRD*, *PDE4D* and *PACRG*, were revealed, as were *TMPRSS2* fusions with the *LINC01525*, *FBXO7*, *GTF3C2*, *NTNG1*, and *YPEL5* genes [48]. In addition, the WGS of PCa specimens from 183 patients revealed higher levels of tumor mutational burden, as well as mutational signatures of the cancer driver genes *NCOA2*, *STK19*, *DDX11L1*, *PCAT1*, and *SETBP1* in AA tumors in comparison to EA tumors [49].

Distinct variations in germline mutations within multiple genes were found for PCa in AA patients. In a meta-analysis study of 19378 PCa vs. 61620 control cases in patient cohorts of AA, nine novel PCa vulnerability loci were determined, including a distinct stop-gain variant in the *ANO7* gene [50]. These nine novel loci were integrated with 269 known PCa risk variants into a multiracial polygenic risk score, which demonstrated the potential to stratify the patient risk of developing aggressive PCa [50]. A study conducted at the Center for Prostate Disease Research evaluating germline mutations in DDR genes in 300 AA and 300 EA patients, matched for age and PCa stage, using a WGS approach, profiled all known mutations for DDR genes. A total of 13 out of 46 DDR genes with P/LP mutations were present in both AA and EA patients. Among the genes found to be mutated frequently in AA patients, but not EA patients, were *RAD* family genes (*RAD51*, *RAD54L*, and *RAD54B*) and *PMS2* and *BRCA1* [51].

At the Center for Prostate Disease Research, a discrete difference in *ERG* expression during PCa development was discovered while comparing 91 AA and 91 EA patients matched for age, Gleason sum, and pathologic stage. The assessment of *ERG* status in whole-mount prostatectomy samples found that the occurrence of *ERG*-expressing tumors was notably higher in EA patients than in AA patients (41.9% vs. 23.9%, *p* < 0.0001). Moreover, AA patients with PCa progression tend to be *ERG*-negative [52]. *ERG* rearrangements during PCa progression seem to be associated with *CHD1* and *SPOP* deletions. The evaluation of 2093 prostate cancers by FISH analysis revealed a strong connection between *CHD1* deletion and a lack of *ERG* fusion (*p* < 0.0001). It was shown that the elimination of *CHD1* in vitro obstructs the emergence of *ERG* rearrangements due to interference with androgen receptor-dependent signaling [53]. When comparing the lack of *CHD1* in AA and EA patients using PCa tissue microarrays, it was discovered that *CHD1* deletion was three times more frequent in AA patients, which may have contributed to worse PCa progression in AA men [54]. The targeted sequencing of 720 PCa specimens from CA, AA, and Asian patients has revealed that mutations in *SPOP* are correlated with ERG rearrangement and *CHD1* deletion, with the ranging frequency of *SPOP* mutations across diverse populations being between 4.6% and 14.4% [55].

Another susceptibility region for PCa risk is the 8q24 locus. Genetic data for this region were analyzed from 73,535 PCa EA patients and it was discovered that there are 12 PCa risk loci with germline mutations having implications for PCa risk stratification [56]. An important PCa risk was found to be associated with rs72725854 at 8q24 in AA men while analyzing the family histories of 9052 PCa patients and 8595 control cases. This risk allele was associated with both an earlier age at PCa diagnosis and aggressive disease and augmented in men with a PCa family history (32% of familial cases vs. 23% with no familial history and 12% of controls) [57]. Another AA-specific variant in the 8q24 region, rs7824364, was correlated with increased PCa risk and predictive of positive biopsy in 199 AA men, who were referenced for initial biopsy based on PSA levels (>2.5 ng/mL) and abnormal digital rectal examination. Furthermore, this variant could predict PCa in a group of patients with PSA < 10 ng/mL and normal digital rectal examination [58].

Mutations in several other genes were found to be prevalent in AA populations. A study at the Center for Prostate Disease Research involving 435 PCa patients using WGS, FISH analysis, and SNP arrays discovered that the deletion of *LSAMP* within the 3q13.31 region was notably higher in AA PCa patients and was correlated with rapid disease progression. In addition, the occurrence of inter-chromosomal translocations was remarkably higher in the tumors of AA patients, showing variation in mutations across the prostate tumors of diverse populations [59]. The exome sequencing of 102 AA patients with localized PCa revealed an ancestry-specific loss-of-function mutation in *ERF*, an ETS transcriptional repressor, which occurred in up to 5% of lethal PCa cases, highlighting the importance of genomic screening in minorities [60]. While comparing prostate tumors between EA (113 tumors) and AA (105 tumors) patients, differences were found in the incidence of *ERG* rearrangement (42.5% in CA; 27.6% in AA men), *PTEN* deletion (19.8% in CA; 6.9% in AA patients), *SPOP* mutations (10.3% in CA; 4.5% in AA men), and *SPINK* overexpression (8.2% in CA; 23.4% in AA patients). *SPINK* overexpression was associated with more aggressive PCa development [61]. Somatic mutations in PCa were investigated using both targeted sequencing data from 436 AA men and 3018 EA men as well as publicly available datasets comprising 250 AA men and 611 EA men. Mutations in *ZFHX3*, *KMT2D* truncations, *CCND1* amplifications, deletions in *ETV3*, and *MYC* amplifications in metastatic PCa were more common in tumors from AA men [62]. The PCa risk allele in the *HOXB13* gene, X285K, in a population of 11688 AA patients with PCa was associated with a 2.4-fold increase in OR and was linked to aggressive disease, suggesting a benefit of genetic testing for early PCa screening in patients of African ancestry [63]. The analysis of prostate tissue datasets from AA and EA patients revealed that the interferon-related DNA damage resistance signature occurred twice as often in AA men, was associated with an *IFNL4* germline variant (rs368234815-ΔG), and was linked to decreased disease-free survival [64]. The comparison of whole exome sequencing of AA PCa patient cohorts (n = 960 and n = 747) and 8128 control cases of AA men from the Genome Aggregation Database for germline mutations, showed that two variants, R14Q in *GPRC5C* and R511Q in *IGF1R*, even at low incidence, 0.47–0.53% vs. 0.01% in the control population, had ORs for PCa of 37.46 and 21.54, respectively, underscoring the need for genetic testing in underrepresented populations [65].

## 3. DDR Genes in PCa: BRCA1, BRCA2, BARD1, PALB2, ATM, and CHEK2

Both BRCA1 and BRCA2 act as tumor suppressors and are involved in the maintenance of genome stability, specifically the HR pathway for double-strand DNA repair. Mutations in these genes are responsible for a large portion of inherited breast and ovarian cancers.

**Figure 1:**
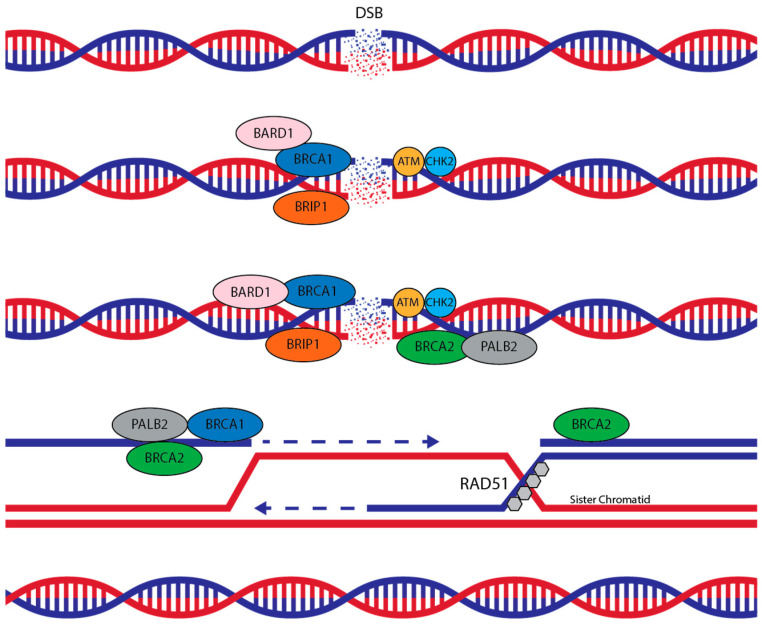
Schematic diagram for DNA double-strand break (DSB) repair by homologous recombination: BRCA1, DNA-repair-associated gene; BRCA2, DNA-repair-associated gene; BRIP1, BRCA-interacting DNA helicase 1; BARD1, BRCA1-associated RING domain 1; PALB2, partner and localizer of BRCA2; ATM, serine/threonine kinase; CHEK2, checkpoint kinase 2; RAD51, RAD51 recombinase.

Proteins involved in HR are products of multiple genes, including *BRCA1*, in close interaction with DNA damage sensor BARD1, RAD51 recombinase, and BRCA2 in association with the PALB2 complex, as well as RAD52, RAD54, and RAD51 paralogs among several other proteins. Encoded proteins of *ATM* and *CHEK2*, on the other hand, are involved in cell cycle checkpoint signaling pathways that are required for genome stability. A putative schematic diagram for HR is outlined in Figure 1.

A histopathological study involving EA men carrying *BRCA1/2* P/LP variants revealed Gleason scores that were significantly higher than in non-carriers. In addition, *BRCA1/2* mutation carrier status was correlated with advanced PCa [66]. The incidence rate of *BRCA1* vs. *BRCA2* PCa and other cancers was investigated through the Consortium of Investigators of Modifiers of *BRCA1/2* (CIMBA), comprising data from 6902 men worldwide (33 countries). The *BRCA2* P/LP variant carrier status was associated with a higher risk of cancers including PCa [67]. A population of 419 EA patients diagnosed with metastatic PCa were tested for germline mutations in DDR genes. Among *ATM*, *BRCA1*, *BRCA2*, and *PALB2* carriers, P/LP variants in *BRCA2* exhibited higher penetrance and were connected to decreased survival (17.4 months vs. 33.2 months, hazard ratio of 2.11, *p* = 0.033) in metastatic PCa [68]. In a study examining 50 metastatic PCa specimens using NGS to analyze 1360 amplicons within 24 HR repair genes, it was found that P/LP in *BRCA2* had the highest occurrence, with a rate of 14%, followed by *ATM* (12%) and *BRCA1* (6%) [69]. A systematic review and meta-analysis investigated the occurrence rate of germline and somatic *BRCA1/2* mutations in metastatic, metastatic castration-resistant, and any-stage PCa. The rates of *BRCA1* germline and somatic mutations were similar in metastatic, metastatic CRPC and any-stage PCa. However, germline and somatic *BRCA2* mutations were significantly increased in all three categories: 3.25% and 6.29% of any-stage PCa, 4.51% and 10.26% of metastatic PCa, and 3.90% and 10.52% of metastatic CRPC patients, respectively [70]. The frequency of *BRCA1/2* germline mutations was additionally examined in 1240 PCa patients, including 30% AA men, using targeted sequencing. Specimens were staged as localized PCa (T2, *N* = 935), advanced PCa (50% T3–4, *N* = 189), and metastatic PCa (*N* = 116). The rates of *BRCA1* mutations were similar in different stages and ancestries, but *BRCA2* showed a significant increase in advanced and metastatic PCa groups regardless of ancestry, with AA patients having more VUS in *BRCA1/2* than EA men (4.6% vs. 1.6%) [71]. Germline P/LP variants in 306 DDR genes were analyzed in a 764-patient cohort of lethal and indolent PCa in both AA and EA patients. It was discovered that cases of lethal PCa were more likely to carry a P/LP variant in DDR genes compared with indolent PCa (18.5% vs. 9.6%), with the *BRCA2* gene being the most frequently mutated [72]. HRD score analysis was used to measure HRD-related PCa risks and to compare germline mutations in *BRCA2*, *ATM*, and *CHEK2* in three patient cohorts from the Cancer Genome Atlas, PROGENE, and Johns Hopkins University, with the highest HRD score being in *BRCA2* cases [73].

Overall, somatic *BRCA1*/*BRCA2* mutations in PCa are more widespread than germline mutations. Both germline and somatic *BRCA2* mutations are more frequent than mutations in *BRCA1*, with the occurrence rate of these mutations being higher in metastatic PCa. While mutations in *BRCA1* are less penetrant, patients carrying *BRCA2* germline mutations are linked to a significantly higher risk of developing PCa and are more likely to progress to advanced stages of the disease.

Emerging data highlight additional genetic mutations associated with predisposition to PCa and aggressive disease. The targeted NGS of 160,790 specimens from multiple cancers, including PCa, revealed that beyond *BRCA1/2* genes, a strong association of the genome-wide loss of heterozygosity and biallelic mutations with PCa was found for *BARD1*, *PALB2*, and *RAD51* paralogs [74]. The targeted NGS of eight DDR genes, *BRCA1/2*, *ATM*, *BRIP1*, *PALB2*, *CHEK2*, *RAD51C*, and *NBN*, was used to investigate hereditary predisposition to PCa in 462 familial PCa cases. P/LP variants were found in 3.9% of cases with occurrence rates within carriers of 38.9%, 22.3%, 11.1%, 11.1%, 5.6%, 5.6%, and 5.6% for *CHEK2*, *ATM*, *PALB2*, *NBN*, *BRCA2*, *RAD51C* and *BRIP1*, respectively [75]. P/LP variants in *CHEK2* were studied in 150 EA PCa patients and 442 healthy individuals. Patients with P/LP variants in *CHEK2* (2.7%) developed PCa significantly earlier than non-carriers (8.9 years, *p* = 0.02), and some pathogenic variants were associated with an increased risk for aggressive disease [76]. Germline mutation screening for the 20 most common PCa-related genes in an underrepresented 113 AA patients revealed rare pathogenic variants in *CHEK2*, *BRCA2*, *ATM*, *FANCA*, *RAD51C*, and *TP53* associated with early onset and advanced disease [77]. In general, germline and somatic mutations in non-*BRCA1/2* DDR genes are less studied, and though rare, they may predispose patients to early-onset PCa and disease progression.

## 4. Therapeutic PARP Inhibitors: Olaparib, Rucaparib, Niraparib, and Talazoparib

**Figure 2:**
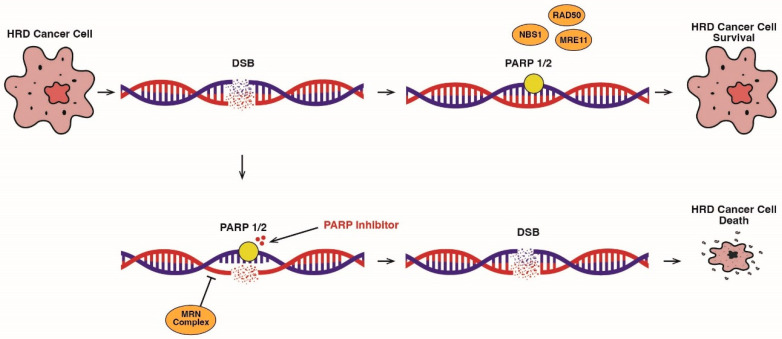
Schematic summary of PARP inhibitor therapy: the main logic of the synthetic lethal approach. HRD—homologous recombination deficiency; MRN—complex containing MRE11, RAD50, and NBS1.

DDR gene mutations are clinically significant targets within precision medicine. PARP inhibitors are small molecule inhibitors that can play a critical role in a synthetic lethal approach, killing HR-repair-gene-deficient cancer cells (Figure 2). The available PARP inhibitors for PCa treatment include olaparib, rucaparib, niraparib, and talazoparib.

In a retrospective study using PROMISE consortium data, 146 patients divided into cohorts by DDR gene mutations and BRCA complex interaction status were administered PARP inhibitors or platinum-based chemotherapy. Cohort A included ninety-four patients with BRCA1/2 mutations, cohort B included forty-five patients with mutations in HR genes that do not interact with BRCA (*ATM*, *CDK12*, *CHEK1/2*, and *FANCL*), and cohort C included seven patients with mutations in HR genes that directly interact with the BRCA complex (*RAD51*, *RAD54L2*, *BARD1*, *PALB2*, *GAN1*, *BRIP1*, and *FANCA*). Patients in cohort A demonstrated significantly longer overall and progression-free survival compared with those in cohort B [78]. Metastatic CRPC patients who were treated with PARP inhibitors (olaparib, n = 116; rucaparib, n = 3; talazoparib, n = 2; veliparib, n = 2), were divided into two groups based on *BRCA* mutation status (*BRCA1* vs. *BRCA2*) and tested for PSA response, progression-free survival, and overall survival. Treatment with PARP inhibitors was associated with a significantly improved response in the group with *BRCA2* mutations vs. the group with *BRCA1* mutations [79]. In a study of metastatic CRPC, the potency of PARP inhibitor therapy was compared in patients carrying *BRCA1/2* mutations and patients carrying *ATM* mutations. It was found that patients with *BRCA1/2* mutations responded significantly better [80]. A systematic review evaluated the efficacy of PARP inhibitors in metastatic CRPC and showed that the most significant benefit of single-agent PARP inhibitors was in patients with mutations in *BRCA1/2* genes, followed by P/LP in *PALB2* and *FANCA* [81].

The efficacy of the PARP inhibitors olaparib, rucaparib, niraparib, and talazoparib was tested in clinical trials. During a phase 2 trial, 50 metastatic CRPC patients who had received docetaxel as prior treatment, and were given abiraterone or enzalutamide, participated in a trial testing the benefit of olaparib treatment. A total of 88% of patients had a response to treatment, including carriers of *BRCA2* (100%) and *ATM* (80%) [82]. In the next phase, metastatic CRPC patients who were treated with abiraterone or enzalutamide but had disease progression were divided into two cohorts—group A (245 patients with *BRCA1*, *BRCA2*, and/or *ATM* mutations) and group B (142 patients with mutations in other DDR genes)—and received olaparib treatment. Interim overall survival rates were higher for patients in group A compared with control group patients, who were taking only abiraterone or enzalutamide (18.5 months vs. 15.1 months) [83]. Upon the completion of the trial, the updated overall survival in both group A and group B remained higher than in the control group (19.1 months vs. 14.7 months and 14.1 months vs. 11.5 months, respectively) [84]. While evaluating different olaparib doses, 400 milligrams twice daily was demonstrated to have a better overall response [85].

In the phase 2 TRITON2 study, the researchers studied 115 metastatic CRPC patients with *BRCA* gene alterations, who progressed after one line of chemotherapy and one or two lines of androgen deprivation therapy. The objective response rate was more than 43.5%, and a higher PSA response rate was recorded in patients with *BRCA2* mutations, overall confirming rucaparib’s antitumor activity [86]. TRITON2-enrolled patients were tested for *BRCA* gene alterations, using central plasma, central tissue, or local genomic testing. Between these three groups, no significant difference was detected in the objective response rate, providing evidence for the primary use of less invasive plasma profiling [87]. During the phase 2 TRITON2 trial, rucaparib efficiency was also tested in 78 patients with alterations in DDR genes other than *BRCA1/2* including *ATM* (49), *CDK12* (15), *CHEK2* (12), and other genes (14). PCa patients with alterations in these genes had limited rates of response to rucaparib [88]. Final data from the TRITON2 trial comprised 277 metastatic CRPC patients grouped by mutations in *BRCA1/2* (one-hundred-and-seventy-two patients), *ATM* (fifty-nine), *CDK12* (fifteen), *CHEK2* (seven), *PALB2* (eleven), and other DDR genes (thirteen). The results demonstrated an objective response rate of 46% for the *BRCA1/2* group, 100% for the *PALB2* group, and 25% for the other-genes group. No objective response was observed in the *ATM*, *CDK12*, and *CHEK2* groups. Overall, the results of this trial confirmed the efficacy of rucaparib to treat metastatic CRPC patients with *BRCA* alterations and selected non-*BRCA* gene mutations [89].

Metastatic CRPC patients who progressed after previous chemotherapy were included in a phase 2 trial for niraparib treatment. Patients with mutations in *BRCA* (142), and non-*BRCA* carriers (81), were prescribed niraparib. The objective response rate was 34.2% for the *BRCA* group compared with 10.6% in non-*BRCA* carriers [90]. In the phase 2 TALAPRO-1 trial, 128 patients carrying mutations in DDR genes, who progressed on abiraterone and/or enzalutamide to metastatic CRPC, were recruited across medical centers in Australia, Austria, Belgium, Brazil, France, Germany, Hungary, Italy, the Netherlands, Poland, Spain, South Korea, the UK, and the USA. Patients who were on a talazoparib regimen, and had mutations in the *ATM*, *ATR*, *BRCA1*, *BRCA2*, *CHEK2*, *FANCA*, *MLH1*, *MRE11A*, *NBN*, *PALB2*, or *RAD51C* genes, demonstrated an objective response rate of 29.8% [91].

In a pooled review of PARP inhibitor efficacy in metastatic CRPC patients with mutations in DDR genes, the lowest hazard ratios were observed for patients having *BRCA1/2*, *CDK12*, or *PALB2* mutations while patients with mutations in *ATM* or *CHEK2* had a limited response to PARP inhibitor treatment [92]. Clinical resistance to PARP inhibitor treatment was reported, and there is evidence that such a phenomenon could be due to secondary mutations in DDR genes in cancer cells, which restore function. Specifically, this was shown for *BRCA2* secondary mutations, which re-establish full length wild-type genes in tumors [93,94] and promote cancer cell survival. On the other hand, a study on PCa familial history in AA patients reported a rare non-synonymous mutation in the *ADPRHL1* gene that leads to PARP activation and cancer cell survival [95]. Such situations warrant the use of PARP inhibitors to suppress overactivated PARP even in the absence of mutations in DDR genes.

Overall, according to the NCCN Guidelines [11], it is recommended to use olaparib/abiraterone, niraparib/abiraterone for *BRCA* mutations, talazoparib/enzalutamide for HR mutations, rucaparib for *BRCA* mutation, and olaparib for HR mutations other than *BRCA* mutations. Olaparib is an option for metastatic CRPC patients who have an HR mutation and whose cancer has progressed on prior treatment with androgen-receptor-directed therapy. Olaparib is the preferred treatment option for patients with a pathogenic mutation (germline and/or somatic) in *BRCA1* or *BRCA2* and is also an option in this setting for patients with other HR gene alterations *(ATM*, *BARD1*, *BRIP1*, *CDK12*, *CHEK1*, *CHEK2*, *FANCL*, *PALB2*, *RAD51B*, *RAD51C*, *RAD51D*, or *RAD54L*). Talazoparib plus enzalutamide is a treatment option for patients with metastatic CRPC and a pathogenic mutation (germline and/or somatic) in an HR gene *(BRCA1*, *BRCA2*, *ATM*, *ATR*, *CDK12*, *CHEK2*, *FANCA*, *MLH1*, *MRE11A*, *NBN*, *PALB2*, or *RAD51C*) who have not yet had treatment in the setting of CRPC.

## 5. DNA Mismatch Repair Genes in PCa, *MLH1*, *MSH2*, *MSH6*, and *PMS2* and Therapeutic Implications: PD-1/PD-L1 Blockade

**Figure 3:**
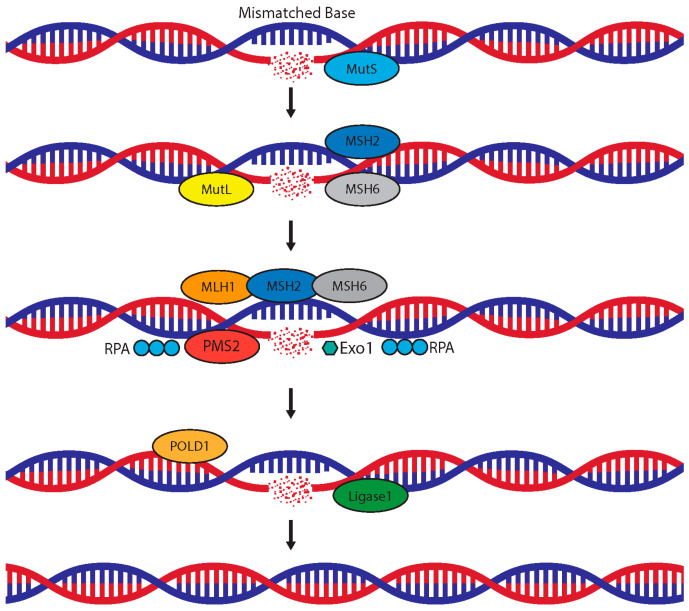
Schematic diagram for DNA mismatch repair: MSH2, mutS homolog 2; MSH6, mutS homolog 6; MLH1, mutL homolog 1; PMS2, endonuclease; RPA, replication protein A; Exo1, Exonuclease 1; POLD1, DNA polymerase delta; Ligase1, DNA ligase 1.

DNA mismatch repair is an important mechanism to eliminate base mismatches and deletions/insertions that could appear during DNA replication and maintain genomic stability. Impairments in the process within microsatellite regions will lead to microsatellite instability and mutations. *MSH2*, mutS homolog 2, and *MSH6*, mutS homolog 6, are tumor suppressors, working in DNA mismatch repair. The encoded protein of *MLH1*, mutL homolog 1, heterodimerizes with mismatch repair endonuclease PMS2 to form MutL. Other proteins involved in MMR are replication protein A, Exonuclease 1, DNA polymerase delta, and DNA ligase 1. The presumed schematic diagram for MMR is presented in Figure 3.

**Figure 4:**
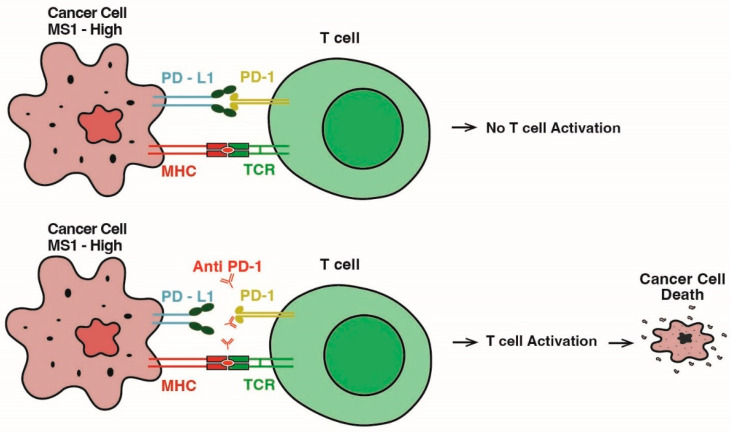
Schematic summary of immunotherapy: Anti-PD1-based T-cell activation in advanced PCa. MSI—microsatellite instability, MHC—major histocompatibility complex, and TCR—T-cell receptor.

Germline mutations in DNA MMR genes, *MLH1*, *MSH2*, *MSH6*, and *PMS2*, are associated with Lynch syndrome and defects in this cellular mechanism will result in genomic instability via the microsatellite hypermutator phenotype and predisposition to the development of multiple cancers including PCa. In a study, the prevalence of Lynch syndrome was evaluated across multiple cancers among 15,045 patients. It was found that Lynch syndrome was detected in 16.3% of patients with microsatellite instability (MSI)-high, 1.9% in patients with MSI-indeterminate, and 0.3% in patients with microsatellite-stable tumors. Though Lynch syndrome is frequently associated with colorectal and endometrial cancers, 50% of MSI-high patients had other tumors including urothelial and prostate cancers [96]. In accordance with this, carriers of mutations in DNA MMR genes were at an increased risk of developing PCa [19]. In a study of MMR-deficient PCa patients, MMR deficiency was found to be secondary to the functional loss of primarily *MSH2*/*MSH6* genes (79%) and *MLH1*/*PMS2* genes (21%) [97]. An immunohistochemical approach to evaluate a link between *MLH1*, *MSH2*, *MSH6*, and *PMS2* expression and clinical data was used in a set of tissue microarrays from 220 radical prostatectomy samples. A significant correlation was found for the loss of MMR gene expression and increased PSA levels, as well as for increased PD-L1 expression in cancer cells [98]. The international prospective IMPACT study evaluated PSA levels and PCa occurrence rate with P/LP variants in DNA MMR genes, *MLH1*, *MSH2*, *MSH6* and *PMS2*, in a cohort of 899 patients predominantly of European ancestry: *MLH1*, 204 patients; *MSH2*, 305 patients; and *MSH6*, 135 patients. The overall PCa incidence rate was 1.9%, but in *MSH2* carriers, it was 4.3%, and in the *MSH6* group, it was 3.0%. In addition, PSA levels were significantly higher in the *MSH2*/*MSH6* groups when compared to control non-carrier patients [99]. Since deficiency in DNA MMR genes is associated with increased PD-L1 expression in tumors, it is potentially targetable with an immunotherapy approach to block PD-1/PD-L1 interaction (Figure 4).

A systematic review analyzing the immunohistochemistry approach to evaluate PD-L1 expression in PCa patients revealed that 29% of acinar tumors, 7% of ductal tumors, and 46% of neuroendocrine carcinomas were positive for PD-L1 expression [100]. A similar study investigating PD-L1 expression during PCa progression discovered that PD-L1 expression was detected in both primary and metastatic sites, and CRPC exhibited higher levels of PD-L1 expression than hormone-sensitive PCa [101]. A related systematic review evaluating PD-L1 expression in patients identified as MMR-deficient demonstrated that such PCa cases showed MSI in 11% of cases, out of which 12% were PD-L1-positive [25]. Pembrolizumab, an immunotherapy agent that blocks the PD-1 receptor on immune cells, and the NCCN-recommended therapy for treating progressed metastatic PCa unresponsive to chemotherapy seemed to work better in combination with abiraterone and prednisone. In the KEYNOTE-365 trial, 103 PCa patients with disease progression were treated with pembrolizumab. The PSA response rate was 56%, the objective response rate was 16%, and the estimated overall survival was 24 months. However, 91% of patients reported adverse effects and one death occurred [102]. According to the NCCN Guidelines, pembrolizumab is an option for certain patients with metastatic CRPC, MSI-high tumors, deficiency in MMR genes, and a tumor mutational burden ≥ 10 mut/Mb. Since the currently available PD-1/PD-L1 based immunotherapy for PCa treatment may cause severe adverse reactions, further related research is necessary to improve clinical outcomes.

The germline and somatic mutation data, with a comparison of Caucasian–American (CA) and African–American patients, are summarized in Table 1 below.

## 6. Conclusions

Germline and somatic mutations in DDR genes represent important factors in PCa development and prognosis, playing essential roles in predisposition to PCa, the growth of localized tumors, and progression to metastatic CRPC. Advances in NGS technology allow whole-genome and deep sequencing, opening an era of precision medicine and personalized medical care. The NCCN Guidelines for PCa recommend germline multigene testing that includes at least *BRCA1*, *BRCA2*, *ATM*, *PALB2*, *CHEK2*, *HOXB13*, *MLH1*, *MSH2*, *MSH6*, and *PMS2*, which are genes involved in both the HR and MMR DNA damage repair pathways. The occurrence rate of P/LP variants in these genes in advanced PCa is increased, and precision medicine approaches are in effect for the treatment of patients carrying such mutations. Diverse populations other than EA men are underrepresented in studying the spectrum and features of germline and somatic mutations in PCa, obligating further research to address disparities and expand new medical care possibilities for these communities. Currently, therapy with PARP inhibitors, such as olaparib, rucaparib, niraparib, and talazoparib, with or without novel hormone interventions, is prescribed for PCa patients carrying mutations in DNA damage HR repair genes. Immunotherapy options include PD-1/PD-L1 blockade with pembrolizumab for MSI-high tumors, deficient DNA MMR genes, or a tumor mutational burden higher than 10 mut/Mb. The further development of personalized medicine focused on DDR genes could improve PCa management and increase overall survival rates for metastatic-castration-resistant disease.

## Figures and Tables

**Table 1:** Based on the cited literature, a brief summary of the analyzed germline and somatic DDR gene mutations is compared between patients with European and African ancestry, where available.

Patient Cohort, *N*	Gene(s)	Somatic, Germline Mutations, %			Reference
		CA	AA	Overall	
Total 200, EA 66.5%	DDR, MMR	63.6			[32]
Total 1351, EA 78%	DDR			P/LP elevated	[33]
Total 1878, EA 70%	*BRCA1/2*, MMR			BRCA1/2—4.6%, MMR—2.8%	[34]
Total 427, EA 44%, AA 56%	DDR, MMR	11.05, VUS 16.32	5.91, VUS 25.32		[37]
Total 3607	DDR, MMR		VUS elevated	DDR 17.2, MMR 1.74	[39]
Total 729, metastatic PCa	*BRCA1/2*, HR, non-HR	13.2, 17.4, 69.4, respectively			[40]
Total 5545 EA; 2775 indolent, 2770 aggressive (467 metastatic)	*BRCA2*, *PALB2*, *ATM*	2.5 aggressive, 0.8 indolent; 0.65 aggressive, 0.11 indolent; and 1.6 aggressive, 0.8 indolent, respectively		Among DDR genes most P/LP/D found in *BRCA2*, *PALB2*, *ATM*	[41]
Total 692; EA 83%, AA 6%, others 11%. Localized, metastatic	16 DDR			11.8 metastatic; 4.6 localized. *BRCA2*—5.3, *ATM*—1.6, *CHEK2*—1.9, *BRCA1*—0.9, *RAD51D*—0.4, and *PALB2*—0.4	[42]
Meta-analysis, EA, metastatic	DDR			Mutations in *NBN*, *BRCA2*, *ATM*, *CHEK2*, and *PALB2* are elevated	[43]
89% EA	DDR, MMR			*CHEK2*, *BRCA1/2*, *ATM*, *NBN1*, *PALB2*, *PMS2*, and *MSH6*—total 8.7	[44]
188 AA, 669 EA, advanced, metastatic	DDR, MMR	36.6 VUS	55.3 VUS		[45]
AA, 11 controls vs. 45 progressed tumors	DDR, MMR		*BRCA2*—27; *ATM*—20, *BRCA1*—13; *PMS2*—11.		[46]
15 high-risk AA tumors	WGS analysis		1.8-fold TMB increase vs. EA	*CCND1* and *MYC* gain. Lower *PTEN* and *PIK3CA* mutation rate vs. EA	[47]
115 AA, 61 EA, advanced tumors	WGS analysis		2-fold increase in structural variations vs. EA	Mutations in AA (*CADM2*, *LSAMP*, *PTPRD*, *PDE4D*, and *PACRG*) vs. EA	[48]
Total 183	WGS analysis		TMB elevated	Mutations in AA (*NCOA2*, *STK19*, *DDX11L1*, *PCAT1*, and *SETBP1*) vs. EA	[49]
Meta-analysis, AA, 19,378 PCa vs. 61,620 controls			Mutation in *ANO7*		[50]
300 AA, 300 CA	WGS analysis, DDR		Elevated mutations in *RAD* family, *PMS2*, and *BRCA1* vs. CA		[51]
91 AA, 91 CA	*ERG*	41.9% tumors	23.9% tumors. PCa progression was mostly *ERG*-negative		[52]
Total 2093, PCa tissue microarrays	*ERG*, *CHD1*		*CHD1* deletion was 3 times more frequent than in CA	*CHD1* deletion was correlated with lack of *ERG* fusion	[53,54]
Total 720	*SPOP*, *CHD1*, *ERG*			*SPOP* mutations were correlated with *ERG* fusion and *CHD1* deletion	[55]
Total 73,535, EA	8q24	12 PCa risk loci			[56]
9052 PCa cases vs. 8595 controls. 199, AA	8q24		Rs72725854, rs7824364		[57,58]
Total 435; WGS, FISH, SNP arrays	*LSAMP*		*LSAMP* 3q13.31 deletion		[58]
Total 102, AA. Localized	WES		*ERF* loss-of-function mutation		[60]
113 tumors, EA. 105 tumors, AA	*ERG* fusion, *PTEN* deletion, *SPOP*, *SPINK*	42.5, 19.8, 10.3, and 8.2, respectively	27.6, 6.9, 4.5, and 23.4, respectively		[61]
436, AA. 3018, EA. Public dataset: 250 AA, 611 EA	Targeted sequencing		*ZFHX3*, *KMT2D*, *CCND1*, *ETV3*, and *MYC*		[62]
Total 11,688, AA	*HOXB13*		X285K is linked to aggressive PCa		[63]
33 AA, 36 EA, 24 non-Hispanic AA, 98 EA, public datasets	*IFNL4*		Rs368234815-deltaG is linked to aggressive PCa		[64]
Total 960 AA, 747 AA. 8128 AA controls	WES		R14Q in *GPRC5C* and R511Q in *IGF1R* are linked to PCa with OR 37.46 and 21.54, respectively		[65]
